# The Week's Work

**Published:** 1910-04-02

**Authors:** 


### The Week's Work.

### HOSPITAL ALMONERS' COUNCIL.

The Hospital Almoners' Council was established three years ago, mainly, we believe, through the efforts of Mr. C. S. Loch, the Secretary of the Charity Organisation Society, who has always been keenly in favour of creating closer bonds of union between the voluntary hospitals and other charitable organisations. The hospitals, not only metropolitan but provincial as well, have gradually learned the value of trained almoners: what is more, it has become increasingly recognised that the almoners' department demands organisation and careful management just as much as any other institutional department. The Royal Free Hospital was the first to see the importance and value of the innovation, and more than fifteen years ago appointed Miss Stewart as almoner. The excellent work that has been done, and is still being done, at the Royal Free by the official almoners is sufficiently well known to need no comment here. The success of that work, largely, has been the inducement which led other institutions to appoint similar officials, and at present there are many large metropolitan hospitals which possess an almoners' department or some variation, embryonic enough it is true in some cases, of the system which the Royal Free introduced. As a means of coping with hospital abuse the almoners' services are very useful. The system of inquiry and report is perhaps not the best system we possess: it is open to the objection that it is a modified form of espionage, with which the fundamental conception of charity, as illustrated in the treatment of the sick, is in direct conflict. These are fine ethical objections which hospital workers are hardly likely to consider, or if considered, to deem sufficiently serious to overshadow the benefits which the system confers upon hospitals by placing some check upon barefaced imposture and flagrant abuse. Nor can they be taken as objections that have weighed with the many hospitals, in the metropolis and in the provinces, which have hitherto declined to appoint almoners. It is much more reasonable to suppose that the governors of such institutions are ignorant of the value of the almoners' work, and that they have no knowledge of what is being done by the departments already in effective operation in other institutions. To such we commend the last annual report of the Almoners' Council which has just been issued, and which gives, succinctly and clearly, a *résumé* of the almoners' state at the present moment.

### WHAT THE ALMONER HAS TO DO.

The Council, which has a strong committee, and has now been actively at work for three years, has for its objects: (1) The selection and training of suitable candidates for posts as hospital almoners; (2) the recommendation of such trained almoners to hospital authorities; (3) the granting of certificates to, and the registration of, trained almoners; and (4) the general development and extension of the work of hospital almoners. Candidates are selected not in accordance with some specified syllabus but on their individual merits, and when one takes into consideration that the work of an almoner is of a particularly delicate nature in many cases, it is evident that no selection committee can be too careful in choosing novitiates. The hospital almoner has perhaps duties which are much less specified than those of any other official connected with a charitable organisation. She—for although there is no reason why a man should not be an almoner, it is a fact that all almoners in these islands are women—is given, more or less, a roving commission, and if not quite a plenipotentiary, is as independent as is possible under existing institutional conditions. She must interview patients and institute inquiries. She must be an almoner in the old sense, the limited sense, in which she figures as an institutional Lady Bountiful, with a blank cheque upon the Samaritan Fund. She must be an unofficial health and district visitor, and (we quote from the Council's report) ensure that the home conditions of the patients be suitable. Finally (we quote once more) she must encourage thrift and urge the necessity of providing for the future by joining clubs and friendly societies. These are onerous duties, and it is unfair to expect an untrained person to be fully cognisant of the orthodox manner in which, for instance, the final clause is to be fulfilled. Perhaps it is unfair to expect any candidate to enter upon the almoner's career with such an extensive programme as the Council lays down, but we imagine that the first two clauses, and they only, would be generally accepted as outlining the scope of almoners' duties. They comprise enough and demand sufficient energy, sufficient character, sufficient skill, and general intelligence on the part of the candidate to make the committee of selection discriminate in choosing. The last two clauses—especially the fourth—demand more than these gifts, for they suppose on the part of the would-be almoner an inherent quality—*Menschenfreundlichkeit*, if you like—of which a selection committee cannot judge, but which the subjects of her inquiries will very keenly appreciate should she possess it, and as sharply recognise the absence should she be deficient in it.

### WHAT THE COUNCIL IS DOING.

The Council has been very successful so far in finding suitable candidates, and it is highly satisfactory to note that almoning appears to be a profession which is becoming increasingly popular. It is an eminently suitable profession for a woman who possesses the necessary judgment, tact, discrimination, and—*Menschenfreundlichkeit*. The scale of remuneration, though not high, is good: the work is undoubtedly interesting, and the co-efficient of general utility is certainly greater than is the case in many other and reputedly more fashionable occupations. During the past year the Council has provided almoners, from among its trained novitiates, for Westminster, the London, Great

Ormond Street, and St. Thomas's Hospitals. An attempt has also been made to organise the work in the provinces, and Miss Mudd, one of the executive committee, has gone to Leeds to deal with the problem there. Three candidates are now training for work in Leeds, and before the end of the present year they will probably be in active work there. The Council has emerged from the experimental stage, and consequently feels the need of making itself more representative of the hospitals and of the various interests involved. Its offices are at Denison House, 296 Vauxhall Bridge Road, S.W., where the Secretary, Mr. E. S. Kemp, will always be glad to welcome anyone interested in the work. The organisation has done such good work, it has shown itself so earnest and active, that it is well worthy any support, from whatever quarter, that may be given to it. We sincerely trust that the closer union between it and the hospitals and charity organisations of whatever kind may be speedily realised.

### PRIVATE CHARITY AND THE STATE.

In the annual report of the Stepney and Mile End Committee of the Charity Organisation Society some interesting observations upon the Reports of the Royal Commission on the Poor Laws are made and the Minority proposals are discussed at some length. The report states that these proposals will "involve an enormous increase in public expenditure and a constant growth in the numbers of those who are dependent on public funds." In the experience of the Charity Organisation Society, the assumption that private charity is no longer of any account cannot for a moment be defended. On the contrary, that experience shows that during the last forty years there has been a striking development of the ingenuity and resources of private philanthropy, "which could not," so the report declares, "have taken place under a system of State control." In recent years voluntary individuals and agencies have shown a much greater tendency to co-operate and to deal with cases of distress in a spirit of thoroughness and discrimination. The Society maintains that while private charity in this country has not yet attained perfection, its general standard is much higher than it has ever before been, while much more regard is being paid to the need of inquiry and the possible effects of relief upon the recipients.

### PROMISCUOUS CHARITY.—THE NEED FOR CO-OPERATION.

In the *Times* of March 26, Mr. C. S. Loch, the Secretary of the Charity Organisation Society, puts forward a plea for closer co-operation between various funds and other charitable organisations. His letter is interesting in so far that it draws attention to the fact that there is, as he justly observes, a state of uncertainty and of ferment in charitable matters at the present time. Funds are being started and organisations founded in more than one quarter. If we possessed the eloquence of the model steward in Gil Blas it would be possible to draw a parallel between this indiscriminate foundation of private charities and the hundred and one little institutions for aiding this or that condition of wretchedness, this or that state of ill-being, that sprang into existence in Germany shortly before the present system of charitable relief was properly organised in that country. Failing such eloquence, and in view of the fact that the matter will soon be threshed out in all parts of the country when the Reports of the Poor Law Commissioners come up for general discussion, we confine ourselves to heartily endorsing Mr. Loch's comments on the present state of affairs. There is great need for consultation and co-operation between the various charities already in being: there is even greater need for consultation with such existing organisations and the philanthropic individuals who desire to embark upon new schemes for the alleviation of distress. The Charity Organisation Society is willing and perfectly able to give such would-be founders of new charities advice and counsel, and to point out on what lines new activities may legitimately and usefully be employed.

### THE DRUG MARKET.

Business in the drug market has hardly recovered from the effects of the Easter holidays, and during the past week there have been few transactions of importance. Cod-liver oil is slightly dearer, but a substantial advance in price does not seem probable. Ipecacuanha is still very scarce, and the high prices recently reached are well maintained. There have, however, been hardly any price alterations of note.

